# *Trichosanthes pericarpium* Aqueous Extract Enhances the Mobilization of Endothelial Progenitor Cells and Up-regulates the Expression of VEGF, eNOS, NO, and MMP-9 in Acute Myocardial Ischemic Rats

**DOI:** 10.3389/fphys.2017.01132

**Published:** 2018-01-17

**Authors:** Nini Fu, Hang Li, Jingchang Sun, Liying Xun, Dongmei Gao, Qitao Zhao

**Affiliations:** ^1^School of Pharmaceutical Sciences, Shandong University of Traditional Chinese Medicine, Jinan, China; ^2^School of Basic Medicine, Shandong University of Traditional Chinese Medicine, Jinan, China

**Keywords:** *Trichosanthes pericarpium*, endothelial progenitor cells, acute myocardial ischemia, vascular endothelial growth factor, endothelial nitric oxide syntheses, matrix metalloproteinase-9

## Abstract

*Trichosanthes pericarpium* (TP) had been widely used to cure patients of cardiovascular disease for 2,000 years in China. This study aims to extend our previous work to explore the mechanism underlying the protective effect of TP on acute myocardial ischemia (AMI). We hypothesized that TP may display its protective effect on AMI by promoting the mobilization of endothelial progenitor cells (EPC) via up-regulating the expression level of vascular endothelial growth factor (VEGF), endothelial nitric oxide syntheses (eNOS), nitric oxide (NO), and matrix metalloproteinase 9 (MMP-9) in AMI rats. To confirm this hypothesis, we treated AMI model rats with intragastrical administration of TP aqueous extract (TPAE), and examined both changes in the number of CEPC, and the expression levels of VEGF, eNOS, NO, and MMP-9 in myocardial tissue and their plasma content in these rats. Rats in each group were randomly divided into seven subgroups. From day 1 to 7 following AMI modeling, rats in these subgroups was sequentially phlebotomized from their celiac artery after being anesthetized by chloral hydrate. We found that, compared with the AMI model rats, in rats treated by TPAE, the CEPC counts, the expression of VEGF, eNOS, NO, and MMP-9 in myocardial tissue and their plasma content all increased more rapidly 7 days after AMI and remained at higher level (*P* < 0.05 or *P* < 0.01). Our results showed that, in AMI rats, the TPAE could significantly promote the mobilization of EPC and up-regulate the expression level of VEGF, eNOS, NO, and MMP-9 in myocardium and their plasma content. Therefore, our results suggest that TAPE may regulate EPC mobilization through up-regulating the expression level of VEGF, eNOS, NO and MMP-9 in the myocardium of AMI rats.

## Introduction

Heart failure due to ischemic heart disease is the leading cause of death worldwide. Human adult hearts have limited capability to generate new vascular endothelial cells, cardiomyocytes, etc. Stem/progenitor cell based-cardiac regeneration offers the basis for repairing the failing hearts following AMI (Murasawa and Asahara, 2008; Laflamme and Murry, 2011).

Endothelial progenitor cells (EPC) are the precursors of vascular endothelial cells and the basis for protecting and repairing the whole body endothelial layers (António et al., 2010). The EPC are mainly stored in the bone marrow (BM) in adult animals at resting state under physiological condition. Only a small fraction of them are able to develop into circulating endothelial progenitor cells (CEPC, EPC in the peripheral blood). During angiogenesis or for repairing injured blood vessel, the EPC have to be mobilized from the BM to the peripheral blood, and then homing into the appropriate sites before they can differentiate into new VEC (Asahara et al., 1999a; Moreno et al., 2009).

Numerous studies confirmed that AMI could induce rapid mobilization of BM EPC, and that EPC participated in the process of revascularization, tissue repair and the recovery of the ischemic myocardium following AMI (Murasawa and Asahara, 2008; Leone et al., 2009; Ye et al., 2012). It was reported that, after AMI or limb ischemia in healthy animals, the EPC proliferate and mobilize from BM rapidly, and the number of CEPC per ml blood increase rapidly in 7 days (Moreno et al., 2009; Povsic et al., 2013; Regueiro et al., 2015). Ischemic tissue releases a number of cytokines, including vascular endothelial growth factor (VEGF), endothelial nitric oxide synthase (eNOS), nitric oxide (NO), and matrix metalloproteinase 9 (MMP-9), etc. (Leone et al., 2009; Ye et al., 2012; Regueiro et al., 2015). These four molecules are known to play key roles in EPC mobilization. VEGF activates MMP-9 via VEGF/eNOS/NO/MMP-9 signal pathway (Asahara et al., 1999a; Aicher et al., 2003; Ling et al., 2012). MMP-9 catalyzes stem cells factor receptor (Kit), and transforms Kit from membrane-bound state (mKit) into soluble state (sKit), which is the stronger activating factor for the EPC mobilization. sKit leads to the transition of BM EPC from the quiescent state to the proliferative state, and enables them to migrate to vascular niche, which favors the differentiation and reconstitution of the stem/progenitor cells (Iwakura et al., 2006). So, all four molecules, VEGF, eNOS, NO, and MMP-9, are indispensable for the mobilization of BM EPC.

**Figure 1:**
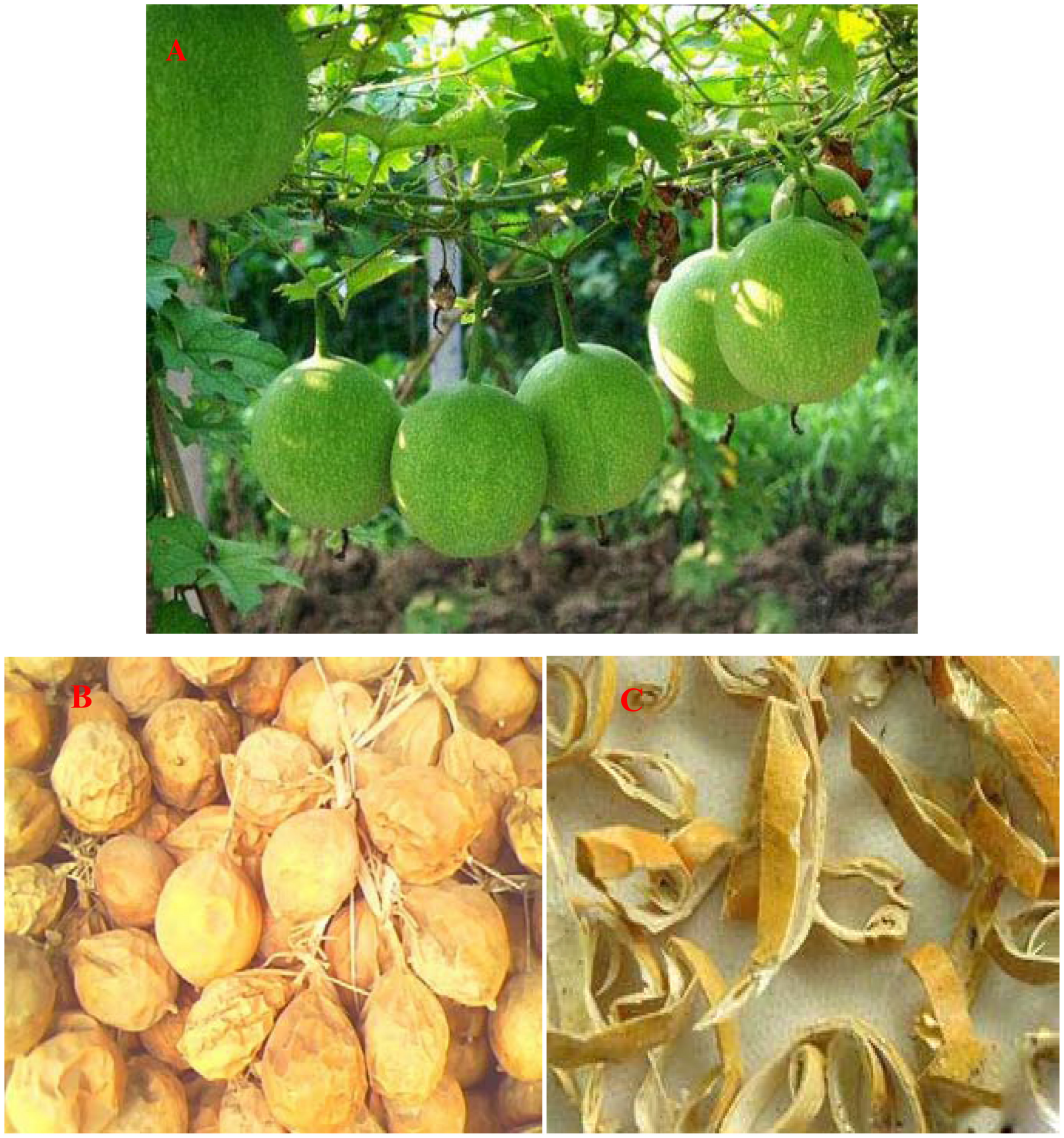
Cucurbitaceae *trichosanthes kirilowii Maxim* and the Chinese herbal medicine *trichosanthes pericarpium* The Chinese herbal medicine *trichosanthes pericarpium* is the dry mature pericarpium of cucurbitaceous *trichosanthes kirilowii Maxim*. It was purchased from Good Agricultural Practice of Traditional Chinese Medicine and Materials (GAP) base of *trichosanthis fructus*, Zhuangke village, Mashan town of Changqing District, Shandong Province, China, and was identified and assessed by Herbal Identification Staff Room, School of Pharmaceutical Sciences, Shandong University of Traditional Chinese Medicine. **(A)** Cucurbitaceous *trichosanthes kirilowii Maxim* and its fruit that shooted in the plantation of *trichosanthis fructus* in SDTCM; **(B)** the dry mature fruit of *trichosanthes kirilowii Maxim;* **(C)** the Chinese herbal medicine *trichosanthes pericarpium*. The voucher specimen (No. 20160807005 **(B)**, No. 20160911003-006 **(C)**) were deposited in the SDTCM.

*Trichosanthes kirilowii* Maxiam (TK, Figure 1A) is a well-known medicinal plant, widespread planted in Shandong, Hebei, Shanxi, Jiangsu and Zhejiang province of China. This herbal medicine has been recorded in literature throughout the history. In TCM, the mature fruit, seed, pericarpium and root tucer of this plant are named *trichosanthis fructus, trichosanthis seed, trichosanthis pericarpium*, and *trichosanthis radix*, respectively. They are frequently used to treat various diseases, and displays marked curative effect (Chinese Pharmacopoeia Commission, 2015).

*Trichosanthes Pericarpium* (TP) is prepared from the dried mature pericarp of TK (Figures 1B,C). Due to its effect on the clearance of heat, the dissipation of phlegm, the amelioration of chest stuffiness, and the regulation of flow of vital energy, TP has been widely used for treating cardiovascular, cerebrovascular, and respiratory diseases for 2,000 years (Yan, 1987; Chinese Pharmacopoeia Commission, 2015). In nowadays, TP and its patent medicine were also widely used to treat patients of coronary heart disease (CHD), angina, hyperlipidemia, and various cardiovascular diseases in China, Taiwan, and Asian countries (Pengfei et al., 2013; Mingzi, 2016; Ren et al., 2016). The rich resource and the minimum side-effect of TP made it much more attractive for further development. However, the mechanism by which TP affect above diseases remains unknown, which makes it difficult to improve the efficacy of TP.

In previous studies, we found that TP displayed distinct protective effect on AMI model rats (Juan et al., 2013; Zhao et al., 2013). The present study aims to extend our previous work, to reveal the mechanism underlying the protective effect of TP on AMI. From a lot of documents and predecessor's researches, we hypothesized that TP may display its protective effect on AMI by promoting the mobilization of EPCs, and up-regulate the expression or secretion of VEGF, eNOS, NO, and MMP-9 in AMI rats. In other words, we think that, these four molecules may be the target of TP. To confirm this hypothesis, we treated AMI model rats with TP aqueous extract (TPAE) through intragastrical administration, and examined the changes in the number of CEPC per ml blood, and the changes in the expression of VEGF, eNOS, NO, and MMP-9 in myocardial tissue and their plasma concentration or activity in these rats.

## Materials and methods

### Reagents

Anti-CD34 monoclonal antibody labeled by PE was purchased from Santa Cruz Co. USA (sc-7324). Anti-vWF antibody labeled by FITC, mouse anti-rat GAPDH monoclonal antibody, goat anti-mouse polyclonal IgG labeled by HRP and rabbit anti-rat MMP-9 monoclonal antibody were obtained from Abcam Co. USA (ab8822, ab9484, ab6789, ab76003). Hemolysin was purchased from BD Co. US (349202). ELISA kits for detecting the plasma level of VEGF, eNOS, NO, MMP-9 was purchased from Bejing Sizhengbo Biological Co. China (CRE0010). A total protein extraction kit was obtained from Applygen Technologies, Beijing, China. RIPA lysis solution was purchased from Beyotime Institute of Biotechnology (P0013B); ECL reagent was obtained from Millipore Co. (WBKLS0500). All other reagents were ultrapure grade.

### Plant materia

The Chinese herbal medicine TP was purchased from Good Agricultural Practice of Traditional Chinese Medicine and Materials (GAP) base of *trichosanthis fructus*, Zhuangke village, Mashan town of Changqing District, Shandong Province, China, and was identified and assessed by Herbal Identification Staff Room, School of Pharmaceutical Sciences, Shandong University of Traditional Chinese Medicine.

#### Preparation of the TP aqueous extract (TPAE)

The air-dried, powdered TP were extracted three times with distilled water at 100°C for 2 h. The combined extracts were filtered and concentrated to reach a concentration of 2 g raw materials/mL. Add 95% ethanol to the concentrated extracts in 2:1 ratio; until the concentration of ethanol in the decoction is about 70%. The total extract was allowed to settle down for 72 h before it was filtered again. The resulting supernatant was concentrated to extractum under reduced pressure and was weighed and stored at 20°C. The extractum was dissolved in 0.9% NaCL (NS) at 10 mg/ml when was intragastrical administered to the rat.

### Animals

The study protocol was followed in accordance with standards and guidelines established by the Guide for the Care and Use of Laboratory Animals formulated by the Ministry of Health, China, and were approved by the Institutional Committee for Animal Care and Use of Shandong University of Traditional Chinese Medicine (approval number: DWSY200710227). All efforts were made to minimize the distress of the animal and the number of animals used in the experiment.

Male wistar rats (220–250 g, SPF) were obtained from the Lu-Nan Animal Experimental Center (SCXK(LU) 20090003, Shandong Province, China), and were housed under 23–25°C RT, 35–65% RH at 12 h light/dark cycle (lights on at 06:00) with free access to food and tap water. Animals were habituated to laboratory conditions for at least 1 week before testing. The health and general behavior of all rats were assessed daily.

### Experimental procedure

The Wistar male rats were first fed for 7 days to adapt the environment. Next, the one hundred and sixty-eight experimental wistar male rats were randomly divided into three groups as follows: Control group (Ctrl; *n* = 56), rats underwent identical surgical procedure, with the exception of coronary artery ligation, and were intragastrical administered with 0.9% NaCL; Model group or acute myocardial ischemia (AMI) group (Isch; *n* = 56), rats underwent coronary artery ligation, and were intragastrical administered 0.9% NaCL; TPAE group (Trich; *n* = 56), rats underwent coronary artery ligation, and were intragastrical administered with TPAE NS solution, according to 2 g per kg avoirdupois.

During the whole experimental period, for rats in different groups, TPAE NS solution and NS were administered once a day respectively. On the 7th day, after the administration (30 min), the rats underwent the left coronary artery ligation. From the 8th day, all the rats go on to be administered with TPAE NS solution or NS until they were executed and phlebotomized.

According to the method described by Zeng et al. (2009), to investigate the mobilization of EPC, rats were executed and phlebotomized at serial time points after establishing AMI models, then the number of CEPC per ml blood in each rats was evaluated by flow cytometric analysis (FCM). In brief, rats in each group mentioned above were randomly subdivided into seven subgroups, namely the 1st−7th subgroup, 8 rats per subgroup. According to the experimental design shown in Table 1, at day 1–7 following AMI modeling, rats in 1–7 subgroups were selected to be phlebotomized from their celiac artery after being anesthetized by chloral hydrate (Table 1) respectively. FCM was performed to quantify CEPC with whole fresh blood sample of each rat.

**Table 1:** Time points of the content of CEPC evaluated by FCM in rats of each subgroup.

**Subgroups**	**1 d**	**2 d**	**3 d**	**4 d**	**5 d**	**6 d**	**7 d**
1st	+						
2nd		+					
3rd			+				
4th				+			
5th					+		
6th						+	
7th							+

*1st−7th, rats in the 1st−7th subgroup, respectively; 1 d−7 d, day 1–day 7 after the coronary ligation, respectively; +, the operation that rats were executed and phlebotomized, and then the FCM was performed to quantify the content of CEPC in these rats*.

### Acute myocardial ischemia model

The rat AMI model was made according to the method described by Zhao et al. (2013). Briefly, rats were anesthetized with an intraperitoneal injection of 10% chloral hydrate (0.3 ml/100 g), then the chest was opened between the left third and the fourth inter costal space, and fixed on the operating table for the surgical procedures. A tracheotomy was performed and an intubation cannula was connected with a volume controlled ventilator. The normal electrocardiogram was recorded via a multi-channel recorder after the electrodes were subcutaneously placed onto the four limbs and connected to an electrocardiograph. The left anterior descending artery (LAD) was ligated by a 6–0 silk suture 1 mm below the tip of the left atrial appendage, and then the heart was repositioned to the chest. Successful ligation was verified by echocardiography and by the color change of hearts.

### Flow cytometric analysis of CEPC

CEPC are defined as cells positive for haematopoietic stem cell markers, such as CD34, and endothelial markers, such as von willebrand factor (vWF) (Asahara et al., 1997; Peichev et al., 2000). So, in this study, the mononuclear cells (MNCs) in blood were positively identified as CEPC by immunofluorescent staining for the both CD34 and the vWF. For assessing the surface markers of cells, whole fresh blood samples anticoagulated by EDTA.2Na were incubated with PE-conjugated anti-CD34 and FITC-conjugated anti-vWF antibodys. Then hemolysin was dissolved in distilled water, and applied to cells at the final concentration 10% (v/v). The CD34^+^/vWF^+^ cells in blood were identified as CEPC.

**Figure 2:**
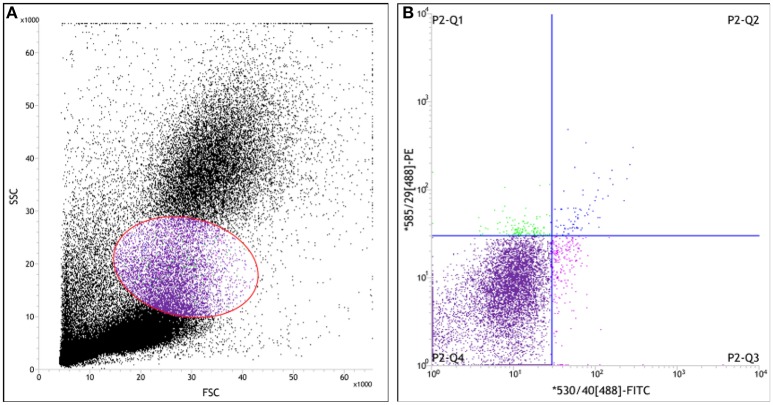
Scatter diagram of leukocyte measured by flow cytometry in the peripheral blood of rat. Whole fresh blood samples anti-coagulated by EDTA.2Na were incubated with PE-conjugated anti-CD34 antibody and FITC-conjugated anti-vWF antibody. Then hemolysin was dissolved in distilled water, and applied to cells at the final concentration 10% (v/v). **(A)** The forward-scatter (FSC) and side-scatter (SSC) diagram, Cells in gate represent the mononuclear cells; **(B)** The fluorescence intensity scatter diagram. MNCs that were positively identified as CEPC by immunofluorescent staining for the PE^+^/FITC^+^ or CD34^+^/vWF^+^ (P2-Q2) represent EPC.

In order to enumerate the CEPC in per milliliter blood, a two-color cytometry analysis of above samples was performed on a Jass cytometry equipped with the four-color option (Becton Dickinson). Appropriate gate analysis was used for the detection of EPC excluding events of different origin, such as non-hematopoietic circulating cells and non-specifically stained events (Figure 2). The data were collected from 50,000 cells for each sample and analyzed with BD FACS^TM^ software (Becton Dickinson, CA).

### Western blots analysis

#### Protein extraction

Rat hearts were removed after animals were phlebotomized from their celiac artery under anesthesia. The LV tissue sample was taken at 2 mm under ligature, frozen in liquid nitrogen, and stored at −80°C before use. The sample was thawed and centrifuged at 20,000 g to fracture the membrane. Total tissue protein was extracted using the total protein extraction kit mentioned above according to manufacturer's instruction.

The protein expression in myocardial tissue of rats was measured by western blot. In brief, the total protein concentration was determined by the BCA protein Assay Kit. Protein was separated by 10% reduced sodium-dodecylsulphate-polyacrylamide gel electrophoresis (SDS-PAGE). Then, protein was transferred to nitrocellulose membranes. The membranes were then blocked by 5% skim milk powder diluted in PBS with 0.05% Tuween (PBST, pH 7.6), and incubated overnight on a rocking platform at 4°C with the antibodies against VEGF, eNOS, MMP-9 (1:2,000), and GAPDH (1:5,000). After that, the membrane was washed with 50 mM PBST three times, 10 min per time, and incubated with the second antibodies for 1 h at room temperature. The membranes were thoroughly washed again, and proteins were visualized by enhanced ECL chemiluminescence solution. Finally, the membranes were exposure with Western Blot Workflow System (Bio RAD). Relative intensities of protein bands were analyzed by Image Lab (Bio RAD).

### Enzyme-linked immunosorbent assay

The plasma level of VEGF, eNOS, NO, and MMP-9 in rats were detected by ELISA. Protocols reference to the manufacturer instruction of these ELISA kits. Whole fresh blood sample above mentioned was centrifugalized 10 min at the speed of 3,000 r/min. The supernate was stored at 4°C. All supernatant samples were tested at the same time after all of rats had been executed and phlebotomized. The plasma level of VEGF, eNOS, NO, and MMP-9 was expressed as ng/Ml, U/mL, or nmol/mL.

### Statistics analysis

Continuous variables were presented as mean ± standard error. Category variables were estimated the statistical significance by one-way ANOVA and *post-hoc* Bonferroni test using SPSS Statistics version 20 (IBM, Armonk, NY, USA). A *p*-value < 0.05 was considered as statistical significance.

## Results

### Effect of TPAE on the mobilization of EPC in AMI rats

**Figure 3:**
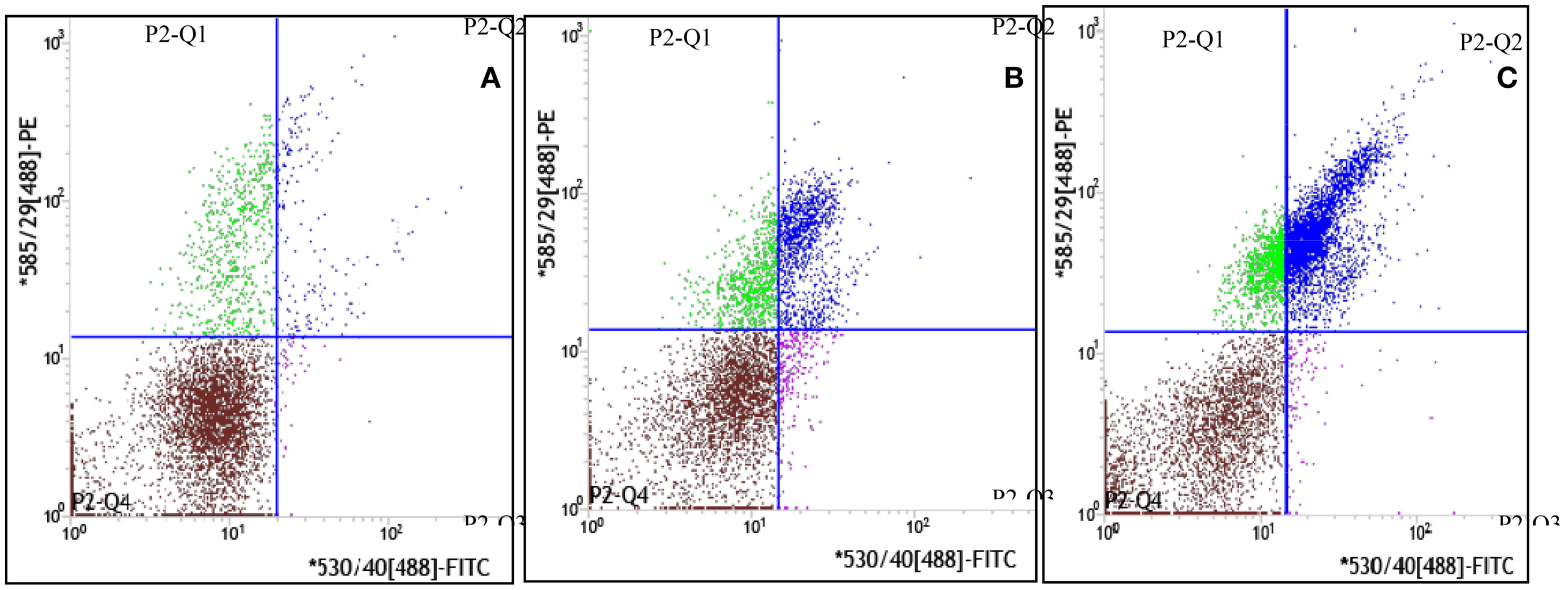
Effect of TAPE on the mobilization of EPC in AMI rats, the fluorescence intensity scatter diagrams of blood samples from the 3rd subgroup rats. Rats in each group were randomly subdivided into seven subgroups, the 1st−7th subgroup. On the 1st−7th day following AMI modeling, rats in 1st−7th subgroups were selected to be phlebotomized from their celiac artery after being anesthetized by chloral hydrate, respectively. FCM was performed to quantify CEPC with whole fresh blood sample of each rat. Cells in gate P2-Q2 (PE^+^/FITC^+^ or CD34^+^/vWF^+^) represent EPC. This is the fluorescence intensity scatter diagrams of blood samples from the 3rd subgroup rats. **(A)** The scatter diagram of blood sample from one rat of the control group; **(B)** The scatter diagram of blood sample from one rat of the model or *isch* group; **(C)** The scatter diagram of blood sample from one rat of the *trich* group.

**Figure 4:**
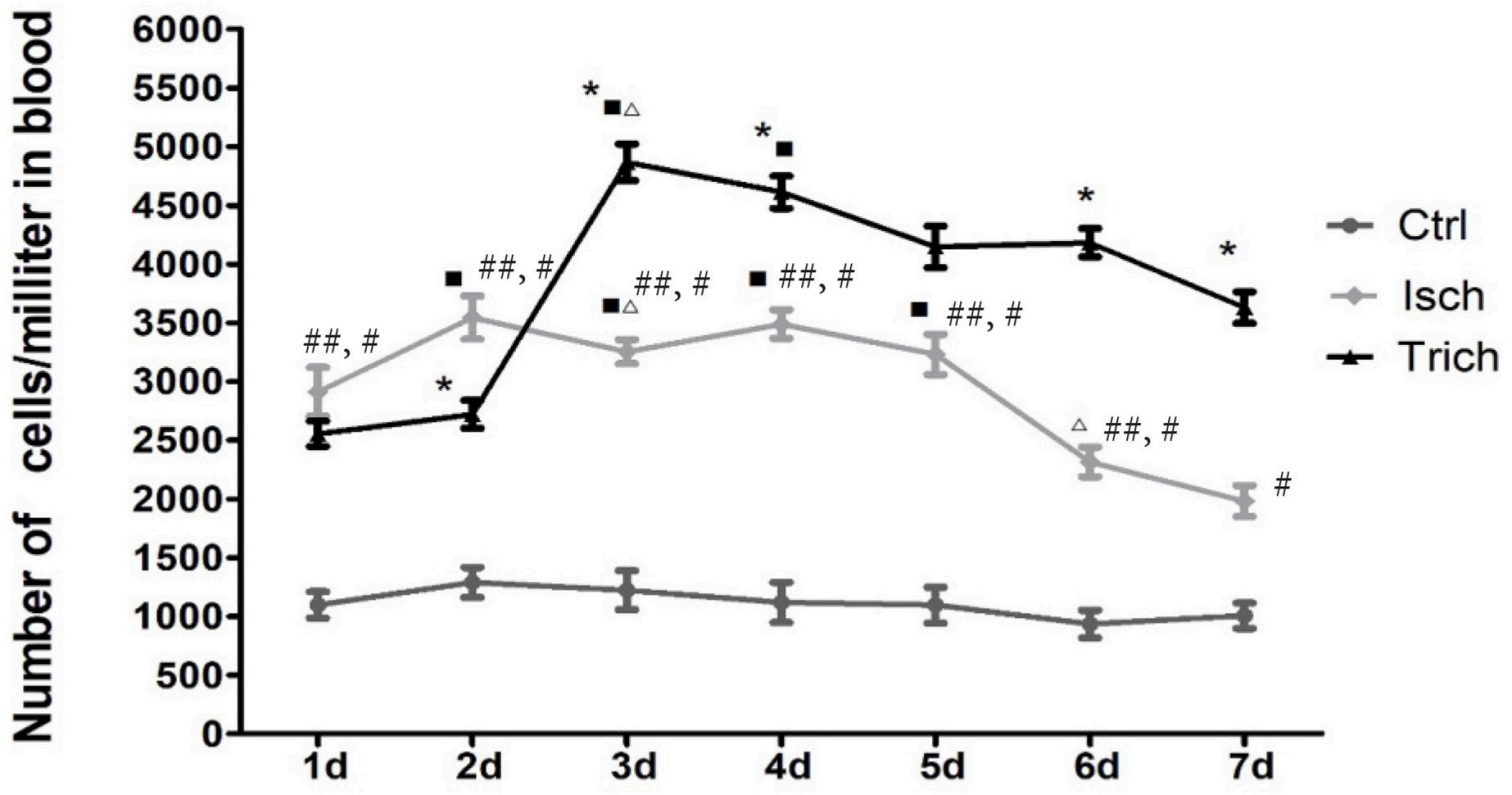
Effect of TAPE on the mobilization of EPC in AMI rats, change in the content of CEPC in each group of rats in 7 days after AMI. Rats in each group were randomly subdivided into seven subgroups, the 1st−7th subgroup. On the 1st−7th day next AMI modeling, rats in the 1st−7th subgroups were selected to be phlebotomized from their celiac artery after being anesthetized by chloral hydrate, respectively. FCM was performed to quantify CEPC with whole fresh blood sample of each rat. Ctrl, Isch, and Trich, represent the CEPC counts per milliliter blood in the rats of control group, the model group and the *trich* group, respectively; 1 d−7 d, represents the 1st−7th day next the coronary ligation or sham operation; # or ##, *P* < 0.05 or *P* < 0.01, compared with the control group; ^*^ or ^**^, *P* < 0.05 or *P* < 0.01, compared with the model group. Each data represents the average of group of 8 rats, and vertical lines indicate the standard error of the mean (SEM).

The dynamic curve was graphed according to the FCM data to show the change on the content of CEPC in individual rats 7 days after AMI. As showed in Figures 3A–C, 4, in control group, the number of EPC in peripheral blood were between 935 and 1290 per milliliter throughout 7 days following sham operation. In the AMI model group, the content of CEPC increased significantly on the 1st day after AMI, and reached its peak value (3,544 ± 183/ml) on the 2nd day. The cell number remained at high level in the next 4 days, and started to decline since the 5th day. In rats received intragastrical administration of TPAE, after the coronary ligating operation, the number of CEPC increased rapidly at the 1st−3rd day, reached its peak value (4868±155/ml) on the 3rd day; this number decreased slowly at the 3rd−6th day. Since the 3rd day after AMI, the content of CEPC in the *trich* group remained at the highest level among three groups (Figures 3A–C, 4, *P* < 0.01).

### Effect of TPAE on the expression of VEGF, eNOS, and MMP-9 in myocardium of AMI rats

**Figure 5:**
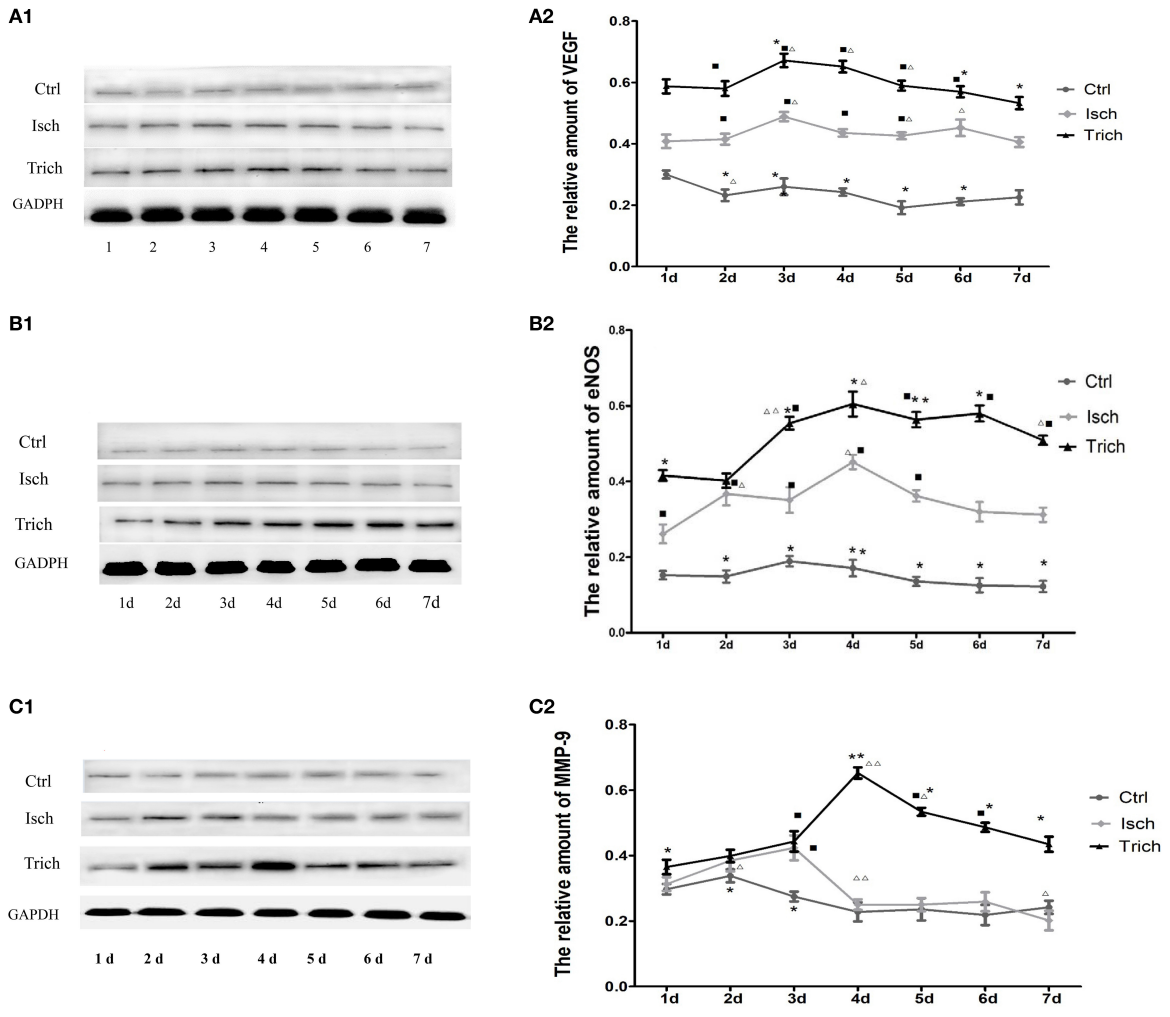
Effect of TAPE on the expression of VEGF, eNOS, and MMP-9 in the myocardium of each group rats in 7 days after AMI. As mentioned above, rats in each group were randomly subdivided into seven subgroups, the 1st−7th subgroup. On the 1st−7th day next AMI modeling, rats in the 1st−7th subgroups were selected to be executed, respectively. Hearts were removed after animals were phlebotomized from their celiac artery under anesthesia. The LV tissue sample was taken at 2 mm under ligature, frozen in liquid nitrogen, and stored at −80°C before use. All myocardial protein samples were detected at the same time after all of rats had been executed and phlebotomized. The expression of VEGF, eNOS, and MMP-9 was analyzed by Western Blot. (**A1,B1,C1)**, Western blot analysis of protein expression of VEGF, eNOS and MMP-9 in the myocardial tissue of each group rats; GAPDH was an internal reference protein for Western blot. **(A2,B2,C2)**, Quantitative Western blot analysis of VEGF, eNOS and MMP-9 in myocardial tissue of each group rats; Ctrl, Isch, and Trich, the control group, the model group and the trich group, respectively; 1 d−7 d, represents the 1st−7th day next the coronary ligation or sham operation; ■, ■■, *P* < 0.05 or *P* < 0.01, compared with the Ctrl group; ^*^, ^**^, *P* < 0.05 or *P* < 0.01, compared with the Isch group. Δ, ΔΔ, *P* < 0.05 or *P* < 0.01, compared with the previous subgroup in the same group. Each data represents the average of group of 8 rats, and vertical lines indicate the standard error of the mean (SEM).

We further detected the expression of VEGF, eNOS, and MMP-9 in myocardium of AMI rats by western blot analysis. As showed in Figure 5A, in control group, VEGF expression level was not changed in myocardium 7 days following sham operation. Compared with the control group, in the model group, the VEGF expression level increased significantly in 7 days after AMI operation and reached peak level on the 6th day, then decreased quickly. Meanwhile, in the *trich* group, its expression level increased more quickly to reach its peak level on the 5th day, and remained the highest level among three groups (Figures 5A1,A2, *P* < 0.01).

As showed in Figure 5B, in control group, no marked change could be observed on the expression of eNOS in the myocardium throughout 7 days following sham operation. Meanwhile, in the model group, since the 1st day following the coronary ligation operation, the expression level of this enzyme in myocardial tissue increased significantly, reached its peak level on the 4th day, and decreased gradually. Compared with the model group, in the *trich* group, the expression level of eNOS increased more rapidly, and decreased more slowly. Moreover, the expression level of this protein remained relative higher than other two groups (Figures 5B1,B2, *P* < 0.05 or *P* < 0.01).

The expression of MMP-9 in the myocardium of rats is showed in Figure 5C. In the control group, the expression level of MMP-9 was stable 7 days after sham operation. Meanwhile, in the model group, its expression level increased rapidly, reached its peak level on the 3rd day following AMI, and decreased quickly to the base level on the 4th day. In the *trich* group, its expression level increased more sharply than the model group, reaching the peak level on the 4th day, and decreased gradually. Since the 4th day, the expression level of MMP-9 was the highest among three groups (Figures 5C1,C2, *P* < 0.05 or *P* < 0.01).

These results suggested that TPAE may significantly up-regulate the expression of VEGF, eNOS, and MMP-9 in the myocardium of AMI rats.

### Effect of TPAE on the plasma level of VEGF, eNOS, NO, and MMP-9 in AMI rats

In order to find out roles of VEGF, eNOS, NO and MMP-9 in the mobilization of EPC in AMI rats and the protective effects of TPAE treatment on these rats, the plasma level of VEGF, eNOS, NO, and MMP-9 in rats were examined by ELISA.

**Figure 6:**
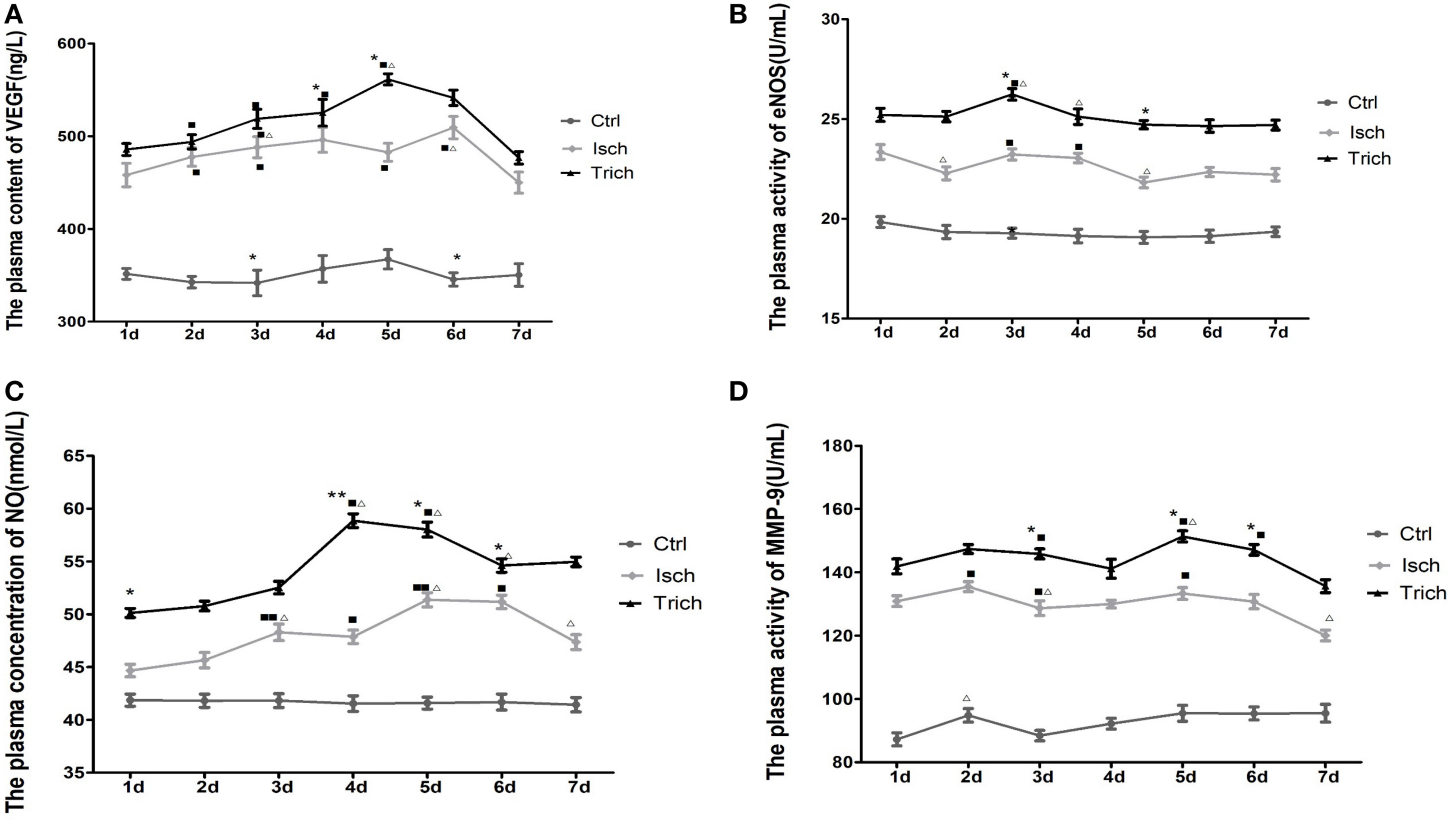
Effect of TAPE on the plasma level of VEGF, eNOS, NO, and MMP-9 of each group rats in 7 days after AMI. Rats in each group were randomly subdivided into seven subgroups, the 1st−7th subgroup. On the 1st−7th day next AMI modeling, rats in the 1st−7th subgroups were selected to be phlebotomized from their celiac artery after being anesthetized by chloral hydrate, respectively. Whole fresh blood sample was centrifugalized 10 min at the speed of 3,000 r/min. The supernatant was stored at 4°C. All samples were tested at the same time after all of rats had been executed and phlebotomized. The plasma level of VEGF, eNOS, NO, and MMP-9 was detected by ELISA. Protocols reference to the manufacturer instruction of these ELISA kits. **(A)** the plasma content of VEGF in each group rats; **(B)** the plasma activity of eNOS in each group rats; **(C)** the plasma concentration of NO in each group rats; **(D)** the plasma activity of MMP-9 in each group rats. Ctrl, Isch, and Trich, the control group, the model group and the trich group respectively; 1 d−7 d, represents the 1st−7th day next the coronary ligation or sham operation; ■, ■■, *P* < 0.05 or *P* < 0.01, compared with the Ctrl group; ^*^,^**^, *P* < 0.05 or *P* < 0.01, compared with the Isch group. Δ, ΔΔ, *P* < 0.05 or *P* < 0.01, compared with the previous subgroup. Each data represents the average of group of 8 rats, and vertical lines indicate the standard error of the mean (SEM).

As showed in Figure 5A, the plasma content of VEGF was relatively steady between 341.78 and 367.31 ng/L throughout 7 days in rats of the control group after sham operation. In the next 7 days after AMI operation, the plasma content of VEGF in the rats of model group increased significantly on the 1st day after AMI, and reached its peak value on the 4th day. This content was higher than that of control groups (449.94–509.3 ng/L). Meanwhile, in the *trich* group, that content was ranged between 476.68 and 561.38 ng/L. After the coronary ligation operation, the plasma content of VEGF began to increase and, reached its peak value on the 5th day; and dropped sharply on the 6th day. Moreover, on the 2nd, 3rd, 4^th^, and 5th day after the AMI, the VEGF plasma concentration was markedly higher than that of model group rats (Figure 6A, *P* < 0.01).

No marked change could be observed on the activity of eNOS (19.07–19.84 U/ml) in the plasma of the control group rats in the next 7 days after the thoracotomy. Meanwhile, in the *Isch* group, the plasma activity of this enzyme fluctuated between 21.82 and 23.35 U/ml, higher than that of the control group. The plasma activity of eNOS in the rats of the *trich* group were at the highest level (24.65–26.24 U/ml) in the same experiments (Figure 6B, *P* < 0.05 or *P* < 0.01).

As showed in Figure 5C, in the control group, in the next 7 days after sham operation the plasma concentration of NO was relatively steady between 41.44 and 41.86 nmol/L. Meanwhile, in the *Isch* group, the NO plasma content increased significantly to, reach its peak value (51.37 nmol/L) on the 5th day, and decreased since the 6th day following AMI operation. The plasma level was significantly higher than that of control group (44.67–51.37 nmol/L) throughout 7 days. In the *trich* group, the plasma content of NO increased gradually to reached its peak value (58.86 nmol/L) on the 5th day, and remained at the highest level among three groups rats (Figure 6C, *P* < 0.05 or *P* < 0.01).

The plasma level of MMP-9 is showed in Figure 6D. In control group, the plasma activity of MMP-9 was relative steady between 87.24 and 95.5 U/mL, and remained at low level in the next 7 days after sham operation. Meanwhile, in the *Isch* group, that activity is significantly stronger than that of control group to reach between 120.05 and 135.49 U/mL. However, in the *trich* group, the plasma activity of MMP-9 increased gradually to reach its peak value on the 5th day, and decreased slowly. Moreover, the activity remained the highest level among three groups in 7 days at 135.65–151.36 U/mL (Figure 6D, *P* < 0.05 or *P* < 0.01).

Our data showed that, TPAE could significantly up-regulate the plasma level of VEGF, eNOS, NO, and MMP-9 in the AMI rats.

## Discussion

Heart failure by AMI is the main cause of death in the world. It has become an unsustainable economic burden for our society. In TCM, TP has been widely used for treating cardiovascular and cerebrovascular diseases for 2,000 years (Yan, 1987; Chinese Pharmacopoeia Commission, 2015). Due to its safety, efficacy and low cost, it is also frequently used to cure AMI, CHD, and hyperlipidemia patients in modern China (Pengfei et al., 2013; Mingzi, 2016; Ren et al., 2016). Our previous studies have showed the significant protective effect of this herbal medicine on AMI model rats (Sun et al., 2013; Zhao et al., 2013). In present study, we further demonstrated that, in the AMI rats, the aqueous extract of TP could promote the mobilization of EPC and notably up-regulate the expression of VEGF, eNOS, and MMP-9.

The relationship between EPC mobilization and cardiac repairment following AMI has been extensively studied. Mounting evidence suggest that AMI is the most accepted acute pathological stimulus for EPC mobilization. Spontaneous mobilization of both HPC and EPC occurs within a few hours after the onset of AMI and remains detectable until after 2 months. These cells are released into peripheral blood and subsequently homed in the myocardium (Ling et al., 2012; António et al., 2014; López-Ruiz et al., 2014; Regueiro et al., 2015). Our present studies also showed that, compared with the control group, within 7 days after AMI in model rats, the circulating EPC counts remained high and peaked on the 4th days following AMI. This finding provides new evidence for the relationships between the mobilization of EPC and AMI.

Although the exact mechanism of EPC mobilization from BM is still poorly understood, it is speculated that it depend on the activation of eNOS, MMP-9 in the presence of several mobilizing factors including VEGF. Asahara had tested the hypothesis that VEGF may modulate EPC kinetics for postnatal revascularization. They observed an increase in CEPC following VEGF administration in mice model (Asahara et al., 1999b). Emerging evidence suggests that the mobilization of EPC after AMI is eNOS-dependent (Iwakura et al., 2006). Mice deficient in eNOS (eNOS^−/−^) showed reduced VEGF-induced mobilization of EPC and increased mortality after myelosuppression. In eNOS^−/−^ mice hind-limb ischemia model, intravenous infusion of wild-type progenitor cells, but not BM transplantation, rescued the defective neovascularization. This finding suggested that the process of progenitor cells mobilization from the BM is impaired in eNOS^−/−^ mice. Mechanistically, MMP-9, which is required for stem cell mobilization, was reduced in the BM of eNOS^−/−^ mice (Aicher et al., 2003). In the diabetic rat model of AMI, decreased circulating EPC was accompanied by reduced expression of phospho-eNOS and MMP-9 (Ling et al., 2012). Many cytokines and active molecules could modulate the kinetics of EPC via either MMP-9-dependent or eNOS-dependent mechanisms, such as estradiol, oxygen/ozone among others (Iwakura et al., 2006; Di Filippo et al., 2010). Our data in this study also showed that, in 7 days after induction of AMI in rat model, the expression level of VEGF, eNOS, and MMP-9 in myocardium and their plasma contents, together with the plasma content of NO, all were up-regulated significantly. This finding provides new insights into the roles of these cytokines in the mobilizations of EPC.

It has been demonstrated that EPC are mobilized to the peripheral circulation in response to AMI, however, the mobilization pattern after AMI remains largely unknown. Regueiro et al measured the EPC levels in AMI patients within 24 h and at 7, 30, 180 days after the acute ischemic event. They found that the number of EPC in peripheral blood was peaked at 30 days post-infarction in these patients (Regueiro et al., 2015). In Shintani'study, in AMI patients, the circulating CD34+ count was quantified on days 1, 3, 7, 14, and 28 after AMI events, and flow cytometry revealed that the cells counts were significantly increased and peaked on day 7 after AMI onset (Shintani et al., 2001). However, in their studies, the daily change on the number of EPC in blood post AMI was not recorded. Our study first characterized the dynamics of EPC mobilization in AMI model rats in the 7 days following the AMI. In our present studies, in order to investigate the mobilization pattern of EPC, rats in each treatment group were further randomly subdivided into 7 subgroups by the date following AMI modeling. The total blood was drawn from the celiac artery of these rats in each subgroups respectively, and the number of CEPC in the whole blood of each rat was quantified by FCM. In our study, rats suffered no hemorrhagic injury before sacrifice except the coronary artery ligation itself. In other studies, it is a common practice to draw blood from the same animal repeatedly, such as drawing blood from orbit, caudal vein, or carotid sinus. These practices lead to local vessel rupture and blood loss, thus activating the mobilization of bone marrow EPC. Moreover, the blood collected from orbit or caudal vein is local to reflect the CEPC level at systemic level. In contrast, our method could maximally exclude other interferences to provide an objective reflection at systemic level. On the other hand, our study can only detect the average CEPC level in the whole circulating blood, rather than the gradient distribution from bone marrow to myocardial ischemia-affected area.

We also investigated the kinetics pattern of plasma VEGF, eNOS, NO and MMP-9 following AMI in this study. Ye's study showed that, in AMI patients during the first 24 h, high VEGF levels were associated with increased EPC levels (Ye et al., 2012). Shintani and Sun observed that, in post-AMI patients, on days 1, 3, 5, 7, 14, and 28 after MI, the kinetics of plasma VEGF was very similar to the kinetics of the EPC (Shintani et al., 2001; Sun et al., 2013). We found that the change patterns of VEGF, eNOS, NO, and MMP-9 plasma level were not similar to the kinetics of the EPC. We speculate that the mobilization of EPC following AMI is regulated synergistically by various factors including cytokines, chemokines, growth factors, and other active molecules. The mean EPC level in peripheral blood might be not associated with the mean plasma concentration or activity of any single specific factor.

In summary, this study aims to extend our previous work, to reveal the mechanism underlying the protective effect of TP on AMI. It is attested by plentiful evidences that all four molecules, VEGF, eNOS, NO, and MMP-9, are indispensable for the mobilization of BM EPCs in AMI. Based on this conclusion of other researchers, we hypothesized that TP may display its protective effect on AMI by promoting the mobilization of EPCs through up-regulating the expression of VEGF, eNOS, and MMP-9 in AMI rats. The finding of present study verified this hypothesis preliminarily. The present study is a part of our serial studies on TP. In further mechanistic studies, the genetic knock-out and the transgenic animals will be used to verify this hypothesis, and to identify the mechanism underlying the beneficial effect of TP.

With the increased understanding of EPC in modern medicine, this type of cell has also gained attention in the field of TCM. In the past decade, studies in this field have confirmed that many kinds of Chinese herbal medicine could improve the function of EPC (Qitao et al., 2014). Understanding the role of Chinese medicine in EPC function may help to develop novel therapeutic strategies in treatment of patients with myocardial damage.

## Author contributions

NF, HL were in charge of making AMI rat model and to design the animal experimental procedure for the investigation of EPCs mobilization. JS was in charge of flow cytometric analysis of endothelial progenitor cells. LX was in charge of enzyme-linked immunosorbent assay and preparation of the TSPP water decoction. DG was in charge of statistics analysis. QZ was in charge of the whole experimental design, the arrangements of researchers and preparation of the paper.

### Conflict of interest statement

The authors declare that the research was conducted in the absence of any commercial or financial relationships that could be construed as a potential conflict of interest.
